# Castleman disease: a case report of a pediatric incidental case in Saudi Arabia

**DOI:** 10.1093/jscr/rjaf712

**Published:** 2025-09-13

**Authors:** Hussain A Alessa, Afnan N Alshayeb, Tahani M Alqurashi, Munira A Suwailem, Ali M Alghamdi, Fahad F Elmokyed, Tahar Yacoubi

**Affiliations:** Department of General Surgery, King Abdulaziz Hospital, King Saud Road, Al Mubarraz District, Alahsa 39182, Saudi Arabia; College of Medicine, Dar Al Uloom University, Airport Road, Al Falah District, Riyadh 13314, Saudi Arabia; Department of General Surgery, King Abdulaziz Hospital, King Saud Road, Al Mubarraz District, Alahsa 39182, Saudi Arabia; Department of Pediatric Surgery, King Abdulaziz Hospital, King Saud Road, Al Mubarraz District, Alahsa 39182, Saudi Arabia; Department of General Surgery, King Abdulaziz Hospital, King Saud Road, Al Mubarraz District, Alahsa 39182, Saudi Arabia; Department of General Surgery, King Abdulaziz Hospital, King Saud Road, Al Mubarraz District, Alahsa 39182, Saudi Arabia; Department of Pathology and Lab Medicine, King Abdulaziz Hospital, King Saud Road, Al Mubarraz District, Alahsa 39182, Saudi Arabia

**Keywords:** Castleman disease, unicentric Castleman disease (UCD), case report, surgery

## Abstract

The most prevalent symptom of Castleman disease, a rare lymphoproliferative condition with an unclear etiology, is a mediastinal nodal mass. While it is quite uncommon in youngsters, it is prevalent in adults. We report a case of a 14-year-old boy who was admitted to the department of surgery for acute right-sided abdominal pain, with suspected acute appendicitis. Preoperative imaging investigation of the abdomen showed an inflammatory appendix as well a large mesenteric mass. Histopathology examination confirmed the diagnosis of acute appendicitis and mesenteric mass unicentric Castleman after resection by laparoscopic approach.

## Introduction

Appendicitis is the most frequent surgical emergency involving abdominal pain in adolescents and young adults, which is defined as inflammation of the vermiform appendix. Currently, either an open or laparoscopic appendectomy is the standard of care for people with this condition [1].

Castleman disease (CD), initially identified by Benjamin Castleman in the 1950s, CD is defined as localized mediastinal lymph node enlargement with substantial capillary proliferation, including follicular and interfollicular endothelial hyperplasia, and a rise in lymphoid follicles with germinal center involution [2].

The co-occurrence of unicentric Castleman disease (UCD) and severe acute appendicitis is extremely uncommon. A mesenteric tumor that was subsequently determined to be UCD was discovered accidentally. In atypical presentations of common illnesses such as appendicitis, the case illustrates the importance of comprehensive surgical exploration and histological examination.

## Case presentation

A 14-year-old boy with a known G6PD deficiency complained of acute, recurring abdominal pain in the right lower quadrant (RLQ). He was diagnosed with gastroenteritis elsewhere after experiencing a similar episode a week prior to admission, which was characterized by dull, widespread discomfort accompanied by nausea and vomiting. His current pain becomes more persistent and colicky. He claimed losing 5 kg of weight in the last few months, but denied having a fever, gastrointestinal bleeding, or systemic symptoms. No family or personal history of cancer or inflammatory bowel disease (IBD).

On physical examination, the patient was alert, oriented, and not in distress. Vital signs were stable, with a blood pressure of 133/64 mmHg, heart rate of 81 bpm, respiratory rate of 20 breaths per minute, temperature of 36.6°C, and oxygen saturation of 99%. Abdominal examination revealed a firm, mobile, non-tender 4 × 4 cm mass in the left lower quadrant with minimal RLQ tenderness on deep palpation. There was no palpable lymphadenopathy, hernias, or rebound tenderness observed.

Laboratory investigations revealed a hemoglobin level of 116 g/L, white blood cell count of 12.9 × 10^9^/L, and elevated C-reactive protein at 121.9 mg/L. Creatinine was within normal limits at 62 μmol/L. Lactate dehydrogenase 280 U/L.

**Figure 1:**
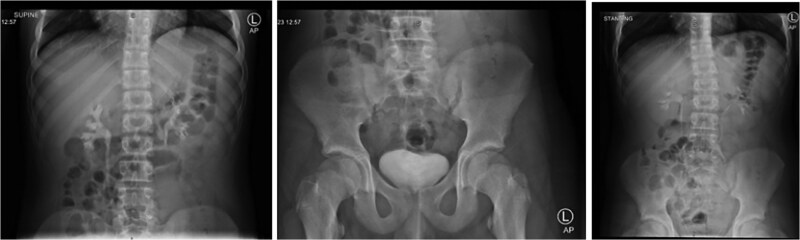
Abdomen/KUB X-ray: Revealed no evidence of intestinal obstruction or pneumoperitoneum in this study.

**Figure 2:**
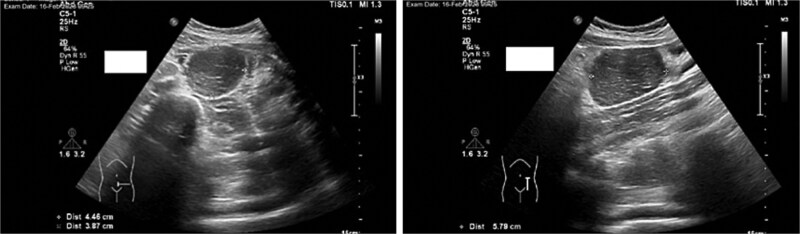
An abdominal ultrasound, which showed a hypoechoic mass in the left lower abdomen*,* and minimal free fluid.

**Figure 3:**
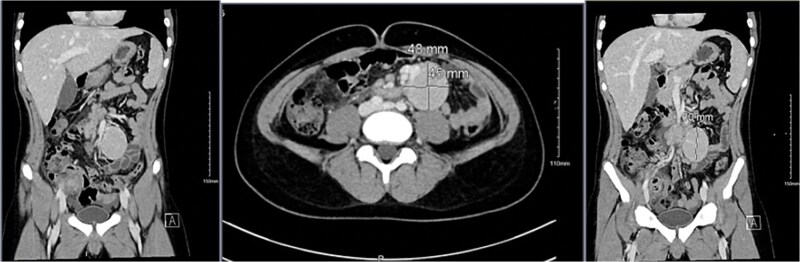
A CT scan of the abdomen revealed an appendicular abscess measuring ⁓40 mL with an associated appendicolith and a soft tissue mesenteric mass measuring 6 × 4 cm, accompanied by enlarged mesenteric lymph nodes.

Imaging studies included abdomen/KUB X-ray was unremarkable (Fig. 1). An abdominal ultrasound, which showed a hypoechoic mass in the left lower abdomen and minimal free fluid (Fig. 2). A computed tomography (CT) scan of the abdomen revealed an appendicular abscess and a soft tissue mesenteric mass measuring 6 × 4 cm (Fig. 3).

**Figure 4:**
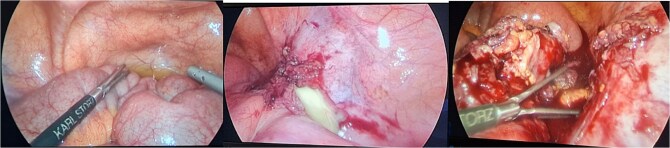
Intra-operative findings included a gangrenous perforated appendix with a right pelvic pus collection.

**Figure 5:**
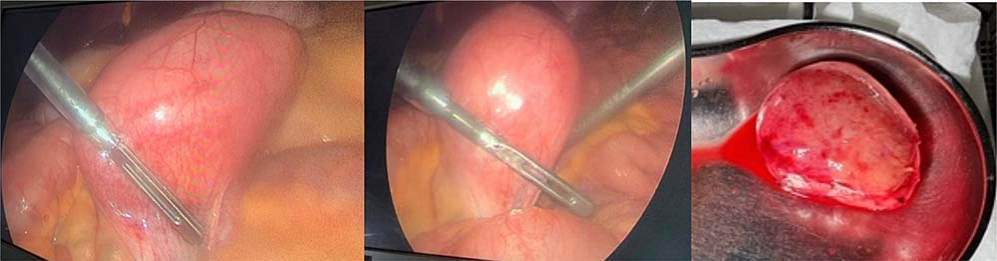
Intra-operative findings, a mesenteric mass measuring 6 × 4 cm was noted ⁓70 cm away from the ligament of Treitz distally.

The patient was admitted, and he underwent an emergency laparoscopic appendectomy with partial cecectomy, mesenteric mass excision, and peritoneal lavage. Intra-operative findings included a gangrenous perforated appendix with a right pelvic pus collection, which was aspirated and sent for culture (Fig. 4). A mesenteric mass measuring 6 × 4 cm was noted ⁓70 cm away from the ligament of Treitz distally and excised (Fig. 5). The surgery was completed without complications.

Histopathological analysis confirmed acute appendicitis with peri-appendicitis and a mesenteric mass consistent with CD, specifically the hyaline vascular variant of UCD.

**Figure 6:**
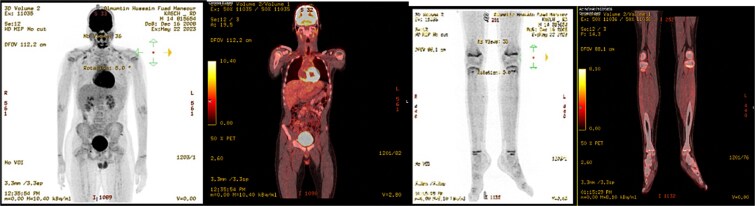
PET scan showed no evidence of hypermetabolic malignancy or lymphoproliferative disease or residual disease.

Post-operative course, patient improved during the admission with smooth post-operative course, discharged in good condition and was started to follow with the hematology clinic after the final histopathological results. A subsequent Pan CT, positron emisssion tomography (PET) scan showed no evidence of metastasis or residual disease (Fig. 6).

## Discussion

CD is an uncommon benign lymphoproliferative condition with an unclear cause. Although it can appear anywhere there is lymphoid tissue, the mediastinum is involved with over 70% of instances, with ˂10% occurring intraabdominally [3], which can be further classified as multicentric, plasma cell, or hyaline-vascular (or angiofollicular) types. CDs are of the hyaline-vascular type, which is a primarily confined lesion with a benign and asymptomatic result [4].

CD is a rare lymphoproliferative condition that can be either unicentric or multicentric in nature. Multicentric Castleman disease (MCD) with multiple lymph nodes, which is linked to human herpesvirus 8, is a systemic illness that most frequently occurs in the context of human immunodeficiency virus (HIV) infection. In contrast, UCD is confined to a single lymph node region and has a good prognosis [5, 6].

There is still no clear pathophysiology for CD. Uncontrolled B-cell proliferation and excessive interleukin-6 release have been linked to CD, according to evidence [7]. CD symptoms are frequently linked to elevated interleukin 6 (IL-6) production [8, 9].

Surgical excision is the primary treatment for UCD, whereas systemic therapy is necessary for MCD. Rituximab is the mainstay, whereas IL-6 inhibitors are becoming more popular. Additional alternatives include antivirals, steroids, and chemotherapy. Management should take into consideration any potential related illnesses like infections or cancers [10–14].

In our rare case, a mesenteric mass incidentally discovered due to appendicular symptoms was completely excised surgically via a laparoscopic approach, and there was no need for any systemic medications.

**Figure 7:**
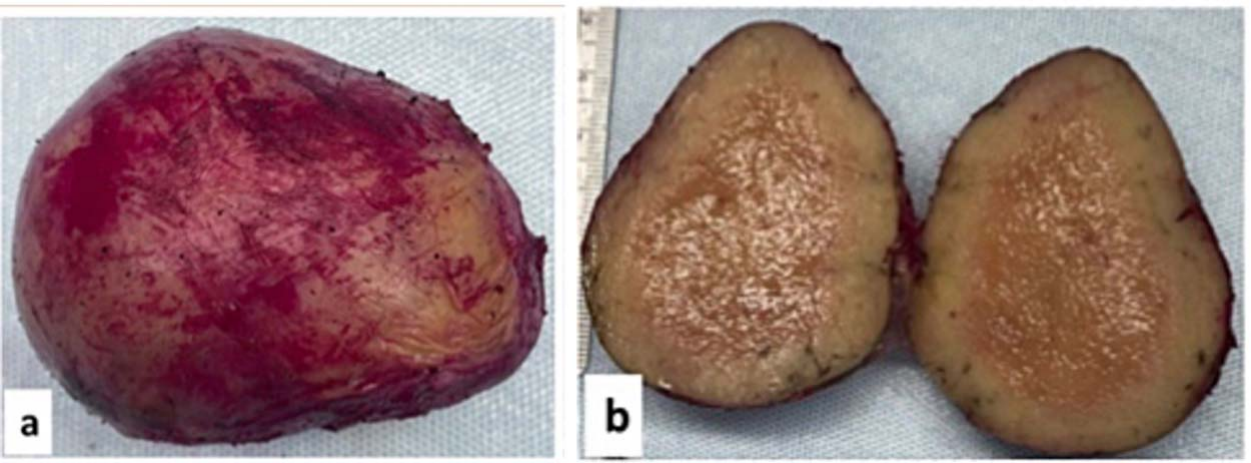
Gross examination (a, b): Mesenteric oval mass (6 cm) with gray homogenous cut surface.

**Figure 8:**
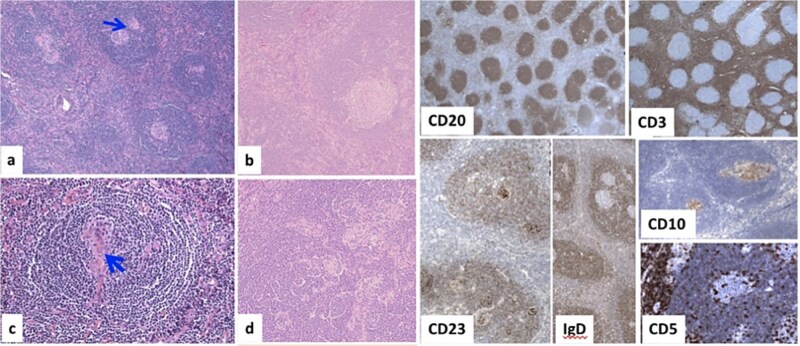
Microscopically, (a) HE × 100: Lymph node showing regressive lymphoid follicles with small and ‘twining’ germinal centers (GC) (arrow). (b, d) HE × 200: Inter-follicular vascular proliferation. (c) HE × 400: Expanded mantle zone, tend to form concentric rings, ‘onion skin pattern’, blood vessels penetrate the GC, ‘lollipop follicle’.

Histopathology examination of our rare case confirmed the diagnosis of acute appendicitis. Gross examination, the mesenteric mass (6 cm) (Fig. 7). Microscopically, it displayed a mixed lymphoplasmacytic infiltration, hyalinized arteries that resembled ‘lollipops,’ and an enlarged lymph node with retrograde follicles. Immunostains revealed polytypic plasma cells, CD20+ B cells, a few CD10+ germinal centers, CD3+/CD5+/CD8+ T cells, a disturbed CD21/CD23 meshwork, and CD31+/CD34+ endothelium. HHV8 stain is negative, and Epstein-Barr virus (EBV) stain showed rare positive cells consistent with past infection (Fig. 8). Serology for HHV8 and HIV is negative.

Children with CD who have a delayed diagnosis have higher morbidity rates because of chronic inflammation. Children are more likely to have UCD (75%) than adults (20%), and pediatric HIV/HHV8-related CD is uncommon. In 30% of instances, lupus is still a differential diagnosis because of autoantibodies. Some pediatric patients had Mediterranean fever gene (MEFV) mutations, suggesting that dysregulated innate immunity may be a contributing factor [15–18].

The patient is for now under surveillance, and no additional treatment was provided.

## Conclusion

In our case, for the suspected involvement of the mesenteric lymph node, which was discovered incidentally due to the appendicular symptoms, we advise proceeding with surgical exploration and excision of the mesenteric mass during the same setting if feasible to decrease postoperative morbidity and mortality and sending the excised mass for histopathological diagnosis.
